# An eight cytokine signature identified from peripheral blood serves as a fingerprint for hepatocellular cancer diagnosis

**DOI:** 10.4314/ahs.v18i2.9

**Published:** 2018-06

**Authors:** Jing Shen, Hua Wu, Ning Peng, Jie Cai

**Affiliations:** 1 Medical Scientific Research Center of Guangxi Medical University, Nanning, 530022, Guangxi, China; 2 Department of Hepatobiliary Surgery, First Affiliated Hospital of Guangxi Medical University, Nanning, 530022, Guangxi Province, China

**Keywords:** Cytokine, peripheral blood, hepatocellular carcinoma, biomarker, diagnosis

## Abstract

**Background:**

Hepatocellular carcinoma is an aggressive disease in Asia and Africa with poor prognosis partially due to lack of disease-specific biomarkers.

**Objectives:**

The aim of this study was to assess the concentrations of different cytokines and chemokines in peripheral blood of patients with hepatocellular carcinoma and identify the potential biomarkers that would help in clinical assessment.

**Methods:**

Profiling of 14 cytokines, chemokines and growth factors was performed in peripheral blood of 78 patients and 78 healthy controls using Bio-Plex Human 15-plex assay kit.

**Results:**

The results showed that patients had significantly higher levels of IL-1β (p=0.034), IL-6 (p=2.13e-06), IL-10 (p=0.013), IL-17A (p=0.017), IL-22 (p=0.00276), IL-25 (p=0.0005), but lower levels of IL-4 (p=0.00341) and IL-33 (p=0.00982) in peripheral blood.

**Conclusion:**

We identified a unique eight-peripheral blood cytokines signature for hepatocellular carcinoma detection. This work will serve as the basis for further studies about the clinical value of peripheral blood cytokines in forecasting prognosis

## Introduction

Hepatocellular carcinoma (HCC) is one of the most prevalent and lethal cancers in Asia and Africa with high mortality rate^1^. Although regular surveillance with alpha fetal protein (AFP) and ultrasound scan is recommended, it is debatable if this strategy improves survival^2^. AFP, the most widely available tumor marker, only has a sensitivity of 41–65% for HCC^3^. New biomarkers for earlier diagnosis of HCC and identification of high risk groups are required.

In the past two decades, several studies have investigated the relevance of cytokines alterations in the multifactorial pathogenesis of hepatocellular carcinoma^4^. Some cytokines mainly present during hepatocellular carcinoma development and can possibly be considered as state markers, such as IL-6 and transforming growth factor-β (TGF-β)^5^,^6^. It has been reported that high levels of pro-inflammatory cytokines in hepatocellular carcinoma might be related to an over-activation of Th17, such as IL-7A, IL-17F and IL-22^7^. However, variations in peripheral blood or plasma cytokines levels might not directly reflect the activity of peripheral blood immune cells. Indeed, peripheral blood and plasma cytokines levels may partly derive from the vessel walls or from other lymphoid or non-lymphoid cells, such as hepatocytes or adipocytes^8^. For these reasons, studying cytokine expression level in peripheral blood might present a more reliable method to investigate the specific activity of immune cells and their degree of activation.

To ascertain whether a peripheral blood cytokine expression signature can distinguish hepatocellular carcinoma from cancer-free controls, we conducted peripheral blood cytokine expression profiling by Bio-Plex Human 15-plex assay kit and extensively evaluated peripheral blood cytokine expression. By statistical analysis, we obtained a profile of eight peripheral blood cytokines, which can serves as a biomarker for hepatocellular carcinoma detection. The correlation between peripheral blood cytokine and hepatocellular carcinoma progression need to be further assessed.

##  Materials and methods

### Study design, patients and control subjects

A multi-stage, case-control study was designed to identify a peripheral blood cytokine profile as a biomarker for hepatocellular carcinoma. Seventy-eight pairs of hepatocellular carcinoma clinical patients (average age:49.5±5.9; Female:69; Smoking:38; Alcohol consumption: 55; HbeAg(+):29) and matched non-tumor samples (average age: 50.8±4.8; Female:65; Smoking:40; Alcohol consumption:53; HbeAg(+):27) were collected at First Affiliated Hospital of Guangxi Medical University from February 2014 and December 2016. The 78 HCC patients were staged according to TNM stage established by Union for International Cancer Control (UICC). All cases had been confirmed by two independent pathologic examinations. Informed consent was obtained from all patients or their guardians and the study was approved by the Research Ethics Committee of First Affiliated Hospital of Guangxi Medical University.

### Quantification of Cytokines, Chemokines

Fresh heparinized blood samples (4 mL) of venous blood which was collected on an empty stomach early morning. The plasma samples (HCC patients and the corresponding controls) were centrifuged at 3000rpm for 10 minutes and then stored at −80°C for further analysis. Subsequently, the samples were analyzed in duplicate using Bio-Plex Human 15-plex assay kit (Bio-Rad Laboratories, Hercules, CA) according to the manufacturer's instructions. The complete list of cytokines (IL-1β, IL-4, IL-6, IL-10, IL-17A, IL-17F, IL-21, IL-22, IL-23, IL-25, IL-31, IL-33, IFN-γ, sCD40L and TNFα) was quantified in these cohorts, and their detection limits and reproducibility were provided in the product manual. The Bio-Plex Protein Array System (Bio-Rad) was employed to distinguish the fifteen distinct sets of fluorescently dyed beads. The detection of 15 cytokine profiling had a high sensitivity and broad dynamic range (0–32,000pg/ml).

### Statistical analysis

Statistical analysis was performed using the Statistical Analysis System software (v.9.1.3; SAS Institute, Cary, NC). Data is presented as the median±SD. Non-parametric Mann-Whitney U test was used to compare the difference of peripheral blood cytokines between the cancer and healthy group. P<0.05 was considered statistically significant.

## Results

### Description and clinical characteristics of the patients

We employed 78 hepatocellular carcinoma patients (diagnosed by two independent pathologic examinations) and 78 healthy people to investigate the potential biomarkers of hepatocellular carcinoma. The clinical characteristics of the patients and healthy samples were represented in Table 1. There was no significant difference in the distribution of smoking (p=0.749), alcohol consumption (p=0.729), age (p=0.059) and gender (p=0.357) between the cancer patients and healthy cases. Among 78 patients, there were 5 patients (6.41%) classified as stages I or II, 56 (71.8%) classified as stages III, 17(21.8%) classified as stages IV, respectively (data not shown). Moreover, elevated levels of AFP (>100ng/ml) were found in 31(39.7%) patients (data not shown).

**Table 1:** Characteristics of the participants

Item	Case (n=78)	Control(n=78)	χ^2^/t	P
Age	49.5±5.9	50.8±4.8	−1.901	0.059
Female	69	65	0.847	0.357
Smoking	38	40	0.103	0.749
Alcohol consumption	55	53	0.120	0.729
TNM stage	I-II	5		
	III	56		
	IV	17		

### Cytokine profiling of peripheral blood in hepatocellular carcinoma and healthy controls

Bio-Plex Human 15-plex assay kit was used to detect the cytokines expression levels in peripheral blood. 15 different cytokines in peripheral blood were assessed in both patient and control subjects. The data showed that there were 14 kinds of cytokines which were detected in peripheral blood (IL_31 was not detected). The result revealed that among the 14 kinds of cytokines in patients with hepatocellular carcinoma, only eight kinds of cytokines have statistical significance (Table 2, p<0.001).

**Table 2:** Comparision of cykotines between paitents vs. healthy control

Cykotines	Group	N	Mean±S.D	t/Z	P
IL_1β	case	78	30.16±7.46	−10.508 *	<0.001
	control	78	12.55±4.32		
IL_4	case	78	36.67±5.34	−10.729*	<0.001
	control	78	67.26±10.93		
IL_6	case	78	3765.71±806.91	−10.782 *	<0.001
	control	78	177.35±29.00		
IL_10	case	78	60.90±10.05	−10.783*	<0.001
	control	78	19.10±3.89		
IL_17A	case	78	5.02±1.36	−11.005 *	<0.001
	control	78	1.08±0.22		
IL_17F	case	78	2.64±0.40	−1.778	0.075
	control	78	2.49±0.81		
IL_21	case	78	31.57±6.79	−0.454	0.650
	control	78	30.99±5.19		
IL_22	case	78	5.14±1.08	9.657 *	<0.001
	control	78	10.45±3.02		
IL_23	case	78	19.02±3.95	0.562	0.575
	control	78	18.62±4.83		
IL_25	case	78	2.10±0.37	10.780 *	<0.001
	control	78	0.69±0.21		
IL_33	case	78	292.52±48.80	−14.744 *	<0.001
	control	78	410.06±50.73		
IFN_γ	case	78	3.07±0.57	−0.724	0.470
	control	78	3.15±0.79		
sCD40L	case	78	1.29±0.46	−1.396	0.165
	control	78	1.39±0.44		
TNF_α	case	78	11.62±3.29	0.585	0.559
	control	78	11.27±4.08		

**Figure 1:**
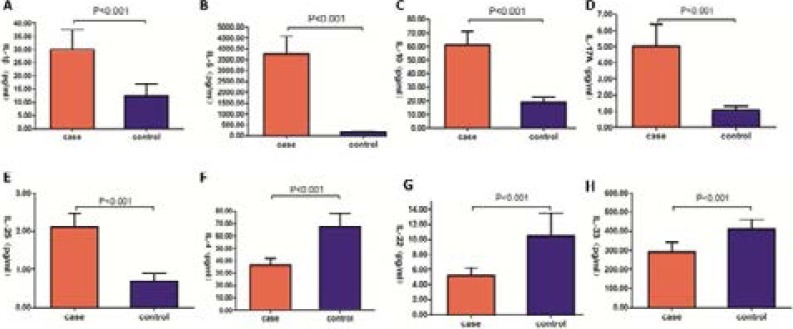
Comparaison of peripheral blood levels of IL-4, IL-1β, IL-6, IL-10, IL-17A, IL-22, IL-25, IL-33 in patients and healthy controls.

From the findings we found that patients with hepatocellular carcinoma had significantly higher peripheral blood levels of IL-1β (30.16±7.46 vs. 12.55±4.32, p<0.001), IL-6 (3765.71±806.91 vs. 177.35±29.00, p<0.001), IL-10 (60.90±10.05 vs. 19.10±3.89, p<0.001), IL-17A (5.02±1.36 vs. 1.08±0.22, p<0.001), and, IL-25 (2.10±0.37 vs. 0.69±0.21, p<0.001). However, the level of IL-4 (36.67±5.34 vs. 67.26±10.93, p<0.001), IL-22 (5.14±1.08 vs. 10.45±3.02, p<0.001), and IL-33 (292.52±48.80 vs. 410.06±50.73, p<0.001) were significant down regulated compared with the healthy controls (Figure 1).

### ROC analysis of eight cytokines

In order to investigate the diagnostic values of the eighty cytokine for hepatocellular carcinoma we performed receiver operating characteristic (ROC) curve analysis (Table 3).

**Table 3:** ROC characteristics of the eighty cytokines

	AUC	Cutoff	Sensitivity	Specifivcity	P value
IL_1β	0.987	20.41	83.33%	98.72%	<0.001
IL_4	0.998	47.08	97.44%	98.72%	<0.001
IL_6	1.0	233.89	100.0%	100.0%	<0.001
IL_10	1.0	28.2	100.0%	100.0%	<0.001
IL_17A	1.0	1.54	100.0%	100.0%	<0.001
IL_22	0.948	7.43	98.72%	83.33%	<0.001
IL_25	1.0	1.24	100.0%	100.0%	<0.001
IL_33	0.769	355.32	88.46%	88.46%	<0.001

**Figure 2:**
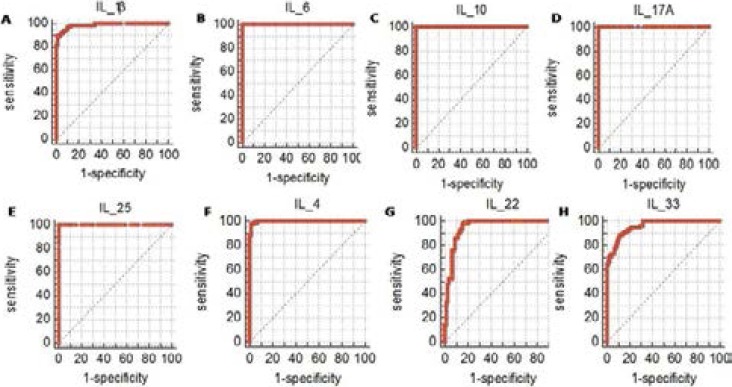
ROC analyses of peripheral blood levels of IL-4, IL-1β, IL-6, IL-10, IL-17A, IL-22, IL-25, IL-33 in patients and healthy controls.

As seen, there were 5 elevated cytokines vs. 3 repressed cytokines among the eight cytokines detected. The ROC characteristic of the five significant increased cytokines were IL-1β (AUC: Area Under the Curve=0.987, P<0.001; Cutoff=20.41, Sensitivity=83.33%, Specificity= 98.72%), IL-6 (AUC=1.0, P<0.001; Cutoff=233.89, Sensitivity=100.0%, Specificity=100.0%), IL-10 (AUC=1.0, P<0.001; Cutoff=28.2, Sensitivity=100.0%, Specificity=100.0%), IL-17A (AUC=1.0, P<0.001; Cutoff= 1.54, Sensitivity=100.0%, Specificity=100.0%), IL-25 (AUC=1.0, P<0.001; Cutoff=1.24, Sensitivity=100.0%, Specificity=100.0%). In addition, the ROC characteristic of the three significant decreased cytokines were IL-4 (AUC=0.998, P<0.001; Cutoff =47.08, Sensitivity=97.44%, Specificity=98.72%), IL-22 (AUC=0.948, P<0.001;Cutoff=7.43, Sensitivity=98.72%, Specificity=83.33%), and IL-33 (AUC=0.769, P<0.001; Cutoff= 355.32, Sensitivity=88.46%, Specificity=88.46%), respectively (Figure 2). The AUC of the eight were all greater than 0.7 which indicated that they represented a high diagnostic value. Furthermore, the high sensitivity and specificity of the 8 cytokines demonstrated their potential role for diagnosis which was consistent with the AUC result.

## Discussion

Hepatocellular carcinoma usually has a poor prognosis mostly due to an advanced stage at the time of diagnosis^9^,^10^. So developing early diagnostic methods at an earlier stage may help to improve prognosis. However, early diagnostic methods with accurate performance and high-throughout biomarkers for hepatocellular carcinoma are not yet available. In the quest for cancer biomarkers, systemic inflammations is frequently highlighted as a potential confounding factor, as cancer development and inflammation have been reported to be associated^11^. In addition, it was known that the identified peripheral blood immune signatures could be considered as snapshots of the immunologic activity in a patient at the time of sampling. Hence, these fingerprints reflect a combination of indirect systemic effects in response to the cancer, as well as factors secreted by the tumor^6^.

On the basis of the notion that immune-regulation is a particular phenomenon in hepatocellular carcinoma, we thus performed the Bio-plex Human cytokines assay to detect the peripheral blood cytokines. The result showed that levels of cytokines IL-1β, IL-6, IL-10, IL-17A, and IL-25 are elevated, but cytokines IL-4, IL-22 and IL-33 were decreased in patients' peripheral blood. Moreover, we employed ROC curve analysis to investigate the potential values of the eighty cytokine for hepatocellular carcinoma. The result revealed that the AUC of the eight were all greater than 0.7 which indicated they represented a high diagnostic value. Furthermore, the high sensitivity and specificity of the 8 cytokines demonstrated their potential role for diagnosis which was consistent with the AUC result. However, there were some limitations in the present study. We identified a unique eight-peripheral blood cytokines signature for hepatocellular carcinoma detection but the cytokines are many and this makes it difficult to be widely used in clinics. The prominent cytokines among these eight cytokines need to be selected in the further studies to provide a wide use for the pathogenesis of HCC. As well, the effect of staging of HCC on the levels of cytokines should be investigated in the further studies.

In sum, we identified a unique eight-peripheral blood cytokines signature for hepatocellular carcinoma detection. This work will serve as the basis for further studies about the clinical value of peripheral blood cytokines in forecasting prognosis.
